# Implementing a Care Coordination Strategy for Children with Medical Complexity in Ontario, Canada: A Process Evaluation

**DOI:** 10.5334/ijic.6073

**Published:** 2022-04-28

**Authors:** Samantha Quartarone, Jia Lu Lilian Lin, Julia Orkin, Nora Fayed, Simon French, Nathalie Major, Joanna Soscia, Audrey Lim, Sanober Diaz, Myla Moretti, Eyal Cohen

**Affiliations:** 1 Child Health Evaluative Sciences, The Hospital for Sick Children, Toronto, Ontario, CA; 2 Institute of Health Policy, Management and Evaluation, University of Toronto, Toronto, Ontario, CA; 3 Division of Paediatric Medicine, The Hospital for Sick Children, Toronto, Ontario, CA; 4 Department of Paediatrics, University of Toronto, Toronto, Ontario, CA; 5 School of Rehabilitation Therapy, Queen’s University, Kingston, Ontario, CA; 6 Department of Chiropractic, Macquarie University, Sydney, AU; 7 Department of Paediatrics, Children’s Hospital of Eastern Ontario, University of Ottawa, Ottawa, Ontario, CA; 8 Department of Pediatrics, Hamilton Health Sciences Centre, McMaster University, Hamilton, Ontario, CA; 9 Provincial Council for Maternal and Child Health, Toronto, Ontario, CA; 10 Clinical Trials Unit- Ontario Child Health Support Unit, The Hospital for Sick Children, Toronto, Ontario, CA; 11 ICES, Toronto, Ontario, CA; 12 Edwin S.H. Leong Centre for Healthy Children, University of Toronto, Toronto, Ontario, CA

**Keywords:** process evaluation, care coordination, complex care, medical complexity, child, family

## Abstract

**Introduction:**

A provincial strategy to expand care coordination and integration of care for children with medical complexity (CMC) was launched in Ontario, Canada in 2015. A process evaluation of the roll-out examined the processes, mechanisms of impact, and contextual factors affecting the implementation of the Complex Care for Kids Ontario (CCKO) intervention strategy.

**Methods:**

This process evaluation was conducted and analyzed according to the United Kingdom Medical Research Council (UK-MRC) process evaluation framework. To evaluate the implementation of the CCKO intervention, a multi-method study design was used, including semi-structured interviews with 38 key informants and 10 families of CMC involved in CCKO. To further understand implementation details across regional sites, provincial-level implementation plans, and process documents were reviewed.

**Discussion:**

Strengths of CCKO included novel collaborations and partnerships between complex care teams, community partners and regional sites. Issues relating to communication and coordination across care sectors created challenges to holistic care coordination objectives. Provincial system fragmentation limited the ability of CCKO to provide seamless care coordination due to the multiple care sectors involved.

**Conclusion:**

This study adds to the understanding of the processes involved in a population-level care coordination intervention for CMC. Lessons learned through CCKO can help facilitate reproducibility and necessary adjustments of the intervention in different settings.

## Introduction

With advances in medical care and technology, there is a growing population of children with medical complexity (CMC), defined as those children with chronic conditions who have functional limitations, elevated service needs and high healthcare utilization [1]. CMC and their families interact with multiple services along the care continuum and frequently experience significant gaps in care due to fragmented services [2]. Prior studies report that structured complex care programs designed to coordinate care for CMC may mitigate healthcare expenditures, enhance access to services and improve child and parental health outcomes [3,4,5,6]. The processes and key components of these care coordination models remain poorly understood, yet this information is essential to implementation successes and failures.

Integration of tertiary care-based complex care programs with community-based health facilities represents a novel approach to caring for CMC across locations, the care continuum and service systems [7,8,9]. Such interventions can reduce family travel by delivering complex care services closer to home and coordinating multiple tertiary care services [7]. To date, limited studies have evaluated the implementation of system-level care integration interventions for complex patient populations.

### Implementation setting

Ontario is Canada’s most populous province with a population of approximately 14.5 million, of whom an estimated 0.67% of all children and youth fulfill criteria for medical complexity, and account for one-third of all child health resource use [10].

Canada’s healthcare system is federally funded and provincially delivered, with single-payer universal coverage for medically necessary healthcare services provided on the basis of need, rather than the ability to pay. There is universal coverage for physician and hospital services and a patchwork of coverage for drugs, homecare, rehabilitation, medical-technology, psychological services and other support services via public funding, private insurance, and out-of-pocket spending [11].

The Provincial Council for Maternal and Child Health (PCMCH) is a provincial organization focused on maternal-child healthcare system improvements and is funded by Ontario’s Ministry of Health. With oversight from PCMCH, the CCKO strategy was launched in 2015 as a five-year demonstration project in response to cumulative recommendations and input from multiple stakeholder groups across Ontario. These included families of CMC, clinicians and administrators from hospitals, primary care providers, home and community care services, children’s developmental and rehabilitation services, community-based support service providers, and school-based healthcare providers.

**Figure 1:**
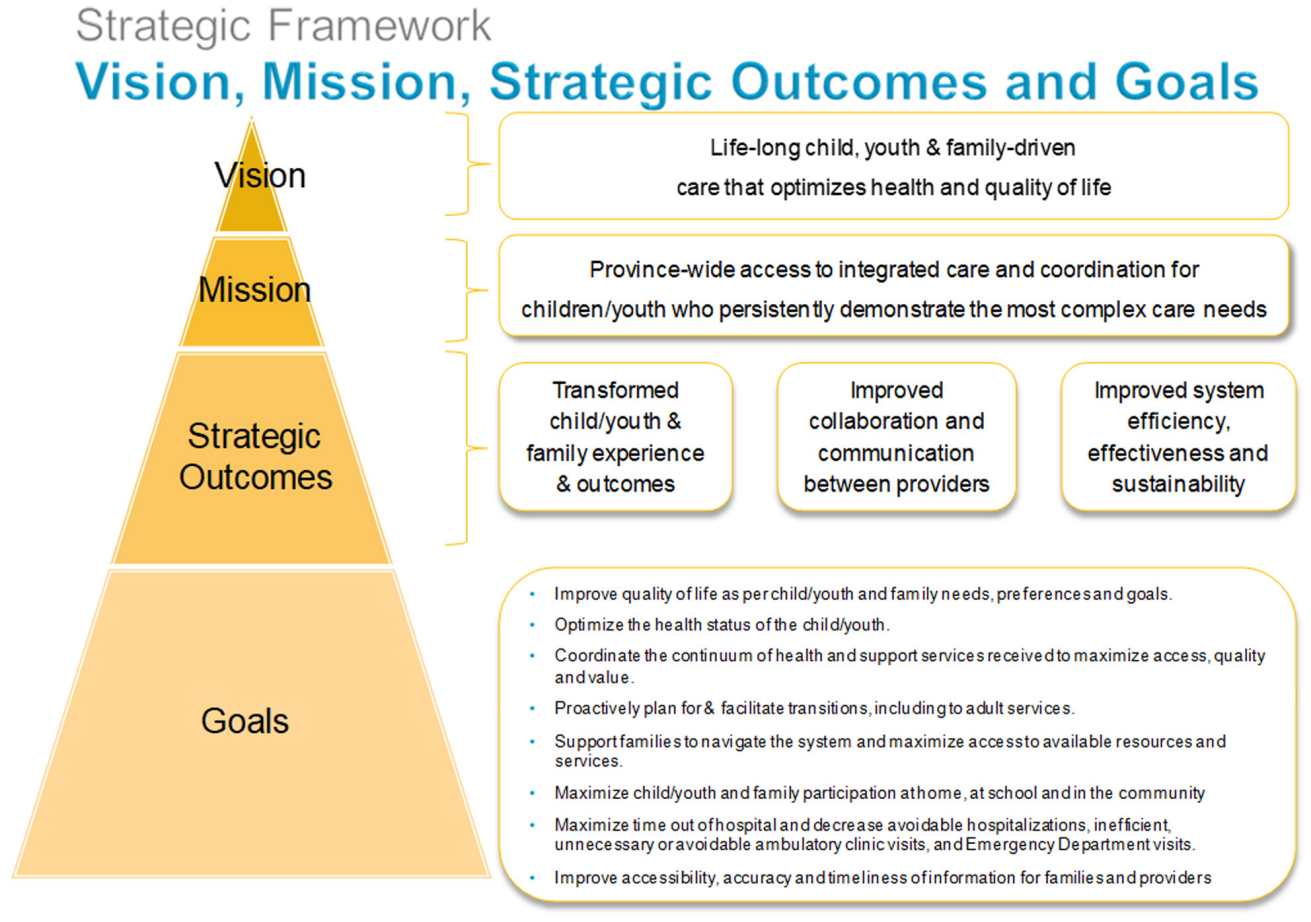
Strategic Framework of Complex Care for Kids Ontario (CCKO) strategy.

The CCKO strategy aimed to expand care coordination and integration for CMC through institutionalizing partnerships among multiple organizations from hospital to home. Building on locally successful tertiary-integrated regional complex care programs, the CCKO strategy set out to expand this hub-and-spoke integrated practice model across Ontario whereby tertiary paediatric hospitals (“hubs”) partner with local hospital and community-based providers (“spokes”) within their geographic catchment area to deliver services for CMC. The strategic framework of the CCKO strategy is shown in ***Figure 1***. The CCKO strategy was implemented through a provincial governance structure enabling ongoing cross-sectoral and cross-organizational collaboration, as well as delegating the responsibility for implementation to four tertiary paediatric hospitals. Details of the CCKO strategy are described in detail elsewhere [12].

To investigate and analyze the processes involved in CCKO implementation, its mechanisms of impact, and the contextual factors affecting implementation success, a process evaluation was conducted according to guidance from the UK Medical Research Council (MRC) process evaluation framework [13]. This MRC framework can help provide insight about why and how complex interventions achieved its outcomes, and how to aid the replication and scale-up of the intervention across settings and populations [14]. Insight was provided by key informants involved in CCKO’s strategic oversight and/or CCKO’s care delivery at complex care clinic(s) and who therefore had first-hand knowledge of the policies, practices, key actors, and relationships specific to their organizational setting.

The aims of this study were: 1) to understand the processes involved in CCKO implementation, including the structures, resources, and processes through which delivery of CCKO was achieved; 2) to investigate the mechanisms of impact and how they led to intervention effects; and 3) to understand the barriers and facilitators influencing the implementation of the CCKO intervention across various settings.

### Implementation processes

**Figure 2:**
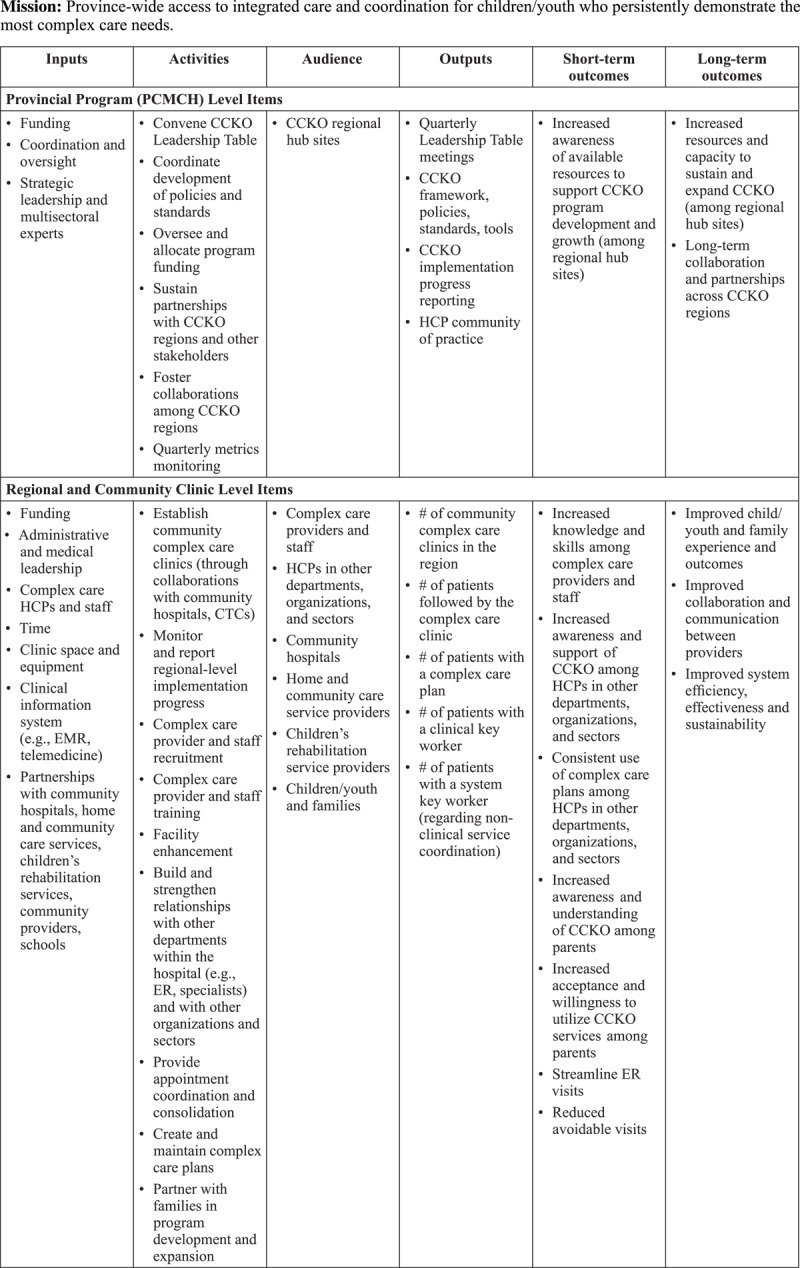
Complex Care for Kids Ontario (CCKO) Logic Model.

Processes relating to the delivery of the CCKO strategy occurred on a i) provincial program level and ii) regional and community clinic level (***Figure 2***).

#### Provincial Program Level

PCMCH, as the organization responsible for oversight and implementation support for the strategy, convened a Leadership Table comprising of leaders from the CCKO regional hub sites, additional representatives from the children’s rehabilitation sector, home and community care services, and parents/caregivers of CMC from across Ontario. The Leadership Table members provided advice to ensure clarity of program standards (operational and performance) and specificity of resource allocation.

The CCKO Leadership Table also monitored implementation progress and recommended adjustments to the implementation approach based on ongoing stakeholder input. A key component of implementation progress monitoring was the quarterly reporting of regional program metrics from the regional hub sites to PCMCH, to facilitate implementation planning, funding allocation and accountability. Developed through the CCKO Leadership Table in partnership with regional hub sites, the standard program metrics included: patient volumes (e.g., current caseload, new intakes, discharges), number of referrals (e.g., received, denied, waitlist), time to next available appointment, and care coordination (e.g., children with a care plan, children with a family doctor/paediatrician). The CCKO Leadership Table reviewed aggregate provincial metrics annually and provided feedback on new metrics to be included, and guidance on implementation strategies based on the data presented.

#### Regional and Community Clinic Level

Implementation of CCKO complex care clinics was led by four regional hub sites, located in Ontario’s major academic centres: The Hospital for Sick Children (Toronto); Children’s Hospital – London Health Sciences Centre (London); McMaster Children’s Hospital – Hamilton Health Sciences (Hamilton); and Children’s Hospital of Eastern Ontario-Ottawa Children’s Treatment Centre (Ottawa). Each regional hub site was responsible for both running a tertiary complex care clinic, and planning and establishing tertiary-integrated complex care clinics with community partners within their dedicated region. In addition, the four regional hub sites were jointly responsible for complex care delivery in Northern Ontario.

At the clinic level, several key actions were essential for ensuring that complex care clinics were able to meet their objectives. Such actions included securing local hospital executive leadership support for complex care clinic establishment, attaining appropriate resources and funding for complex care clinic operations, negotiating clinical space and access to the required equipment to conduct team-based clinic visits with CMC and their families, and building a dedicated allied health team tailored to provide care for CMC.

Within the CCKO care delivery model, care coordination was facilitated by a clinical key worker, typically a nurse practitioner, who supported the complex care team in enacting the coordinated care plan between acute care, primary care, rehabilitation, home and community care. The core team was comprised of the nurse practitioner, home care coordinator and paediatrician, in addition to the administrative assistants that coordinate appointments. Moreover, the allied health professionals, including social workers, dietitians, and pharmacists, were sometimes provided in-kind by hospital and community partners. At each centre where a complex care clinic was established, education and promotion efforts aimed to increase awareness of the program across the organization, as well as to build and strengthen relationships within the organization (e.g., with the emergency department and specialists) and with other organizations and sectors to encourage referrals, collaboration, and appointment coordination. Within the organization, this meant educating staff and trainees to understand the complex care program and its referral criteria and referral process; and outside of the organization, this meant forming relationships with community and rehabilitation care providers to have their representatives attend complex care appointments with the family and the clinical team. Garnering support for the complex care program from across the entire spectrum of care delivery settings for CMC entailed advocating for the program at various community forums (e.g., community paediatrician groups, hospitals and other care organizations that refer to specialty care at the hospital where the complex care clinic resides), in addition to educating community providers about the CCKO complex care delivery model. Moreover, each regional complex care clinic was responsible for capacity building within the community through supporting a hub-and-spoke model where the tertiary nurse practitioner worked in both the tertiary hospital and in community complex care clinics to share knowledge with providers from community hospitals, rehabilitation, primary care and home and community care sectors. Organizational support, academic support, and executive sponsorship were required to support the development of a complex care clinic, in both tertiary and community sites.

## Methods

### Study design

A multi-method study design was utilized to evaluate the implementation of the CCKO intervention, based on information from: i) semi-structured interviews with key informants involved in CCKO implementation; ii) analysis of provincial-level CCKO process documents (e.g., funding reports); and iii) semi-structured interviews with families of CMC who received the CCKO intervention for a minimum of one year. This study received ethics approval from The Hospital for Sick Children Research Ethics Board (REB number: 1000062809).

### Data collection

Semi-structured interviews with key informants who implemented CCKO were conducted in-person or by telephone. The administrative lead of each regional hub site provided a list of healthcare professionals from their region who were involved in CCKO implementation. Potential participants were recruited via email, and all participants provided written informed consent before scheduling an interview. A maximum variation sampling approach yielded a sample of 38 key informants with diversity in profession, care delivery setting, and CCKO implementing region, who were involved with CCKO strategic oversight and/or CCKO care delivery at complex care clinic(s) [15]. An interview guide (Appendix) informed by the MRC process evaluation framework covered topics related to: 1) how key informants understood the CCKO strategy and CCKO complex care model; 2) personal experiences with implementing the CCKO strategy; 3) content of the CCKO model as delivered; and 4) perceived factors that influenced implementation success.

To enrich our understanding of the quality and processes of CCKO implementation across the four implementing regions, provincial-level CCKO process documents from 2015 to 2018 were gathered to complement the analysis. These included regional business plans, regional quarterly metrics, and Leadership Table meeting minutes.

Additionally, parent caregivers of CMC enrolled in the intervention group of the CCKO outcome evaluation were invited to participate in a qualitative interview at one-year follow-up. An interview guide was developed and used to focus on topics related to: family life; family experience with the complex care clinic; family experience with the main healthcare provider in the complex care clinic; family experience with the complex care plan; and suggestions for improvement of the complex care program.

### Data analysis

All interviews were audio recorded and transcribed verbatim by a professional transcriptionist. Initially, the research team read and re-read transcripts to become familiar with the entire dataset. Following this, the data were analyzed using framework analysis [16]. The research team systematically read transcripts line by line and assigned codes according to the MRC process evaluation key components: implementation fidelity, implementation process, mechanisms of impact, and contextual factors [17]. Repeated patterns identified across the dataset were sorted into themes and subthemes. The research team regularly engaged in consensus meetings to discuss the interpretation of qualitative data with the intention to avoid bias, increase consistency and enhance credibility.

Moreover, the contextual factors were analyzed using the Context and Implementation of Complex Interventions (CICI) framework to determine how the contextual factors identified by key informants correspond to the CICI framework domains (i.e., geographical, political, epidemiological, ethical, legal, socio-cultural, and socio-economic) [18]. Within the relevant domains, specific themes emerged from the interviews, which are referred to as sub-domains of the context. Within each sub-domain, we differentiated between the existing elements and required elements discussed by the key informants. Existing elements refer to the components of the context that were present during the design and implementation of the intervention, whereas required elements were those identified by key informants as needed to successfully promote implementation [19].

In addition, the CCKO process documents were used to extract data on implementation fidelity, implementation processes, barriers, and facilitators. Qualitative data on regional complex care program history, state of development, planned and completed activities, and challenges and enablers to CCKO were extracted from regional business plans and Leadership Table meeting minutes. A qualitative data analysis software, NVivo 12 (QSR International), was used to support qualitative data management, coding and analysis.

## Results

Leadership Table members, clinicians, and administrative staff (*n* = 38) involved in CCKO’s strategic oversight and/or care delivery at complex care clinics participated in the study. These key informants completed a brief demographic survey on their age, gender, workplace, role in the CCKO strategy and years of work experience, as summarized in ***Table 1***. In addition, parent caregivers of CMC enrolled in the intervention group of the CCKO outcome evaluation (*n* = 10) participated in the study, and ***Table 1*** also describes their characteristics.

**Table 1:** Characteristics of key informants, parent caregivers and their children with medical complexity.


CHARACTERISTICS OF KEY INFORMANTS	*(N = 38)*	%

Gender		

Female	33	87

Male	5	13

Age (years)		

25–34	5	13

35–44	15	39

45–54	13	34

55–64	5	13

Years of experience in complex care		

Less than 6 months	3	8

6 to 11 months	2	5

1 to 2 years	4	11

3 to 4 years	8	21

5 to 10 years	15	39

11 to 20 years	6	16

Role in CCKO strategy		

Leadership Table member*	11	29

Nurse practitioner	8	21

Physician	6	16

Allied health professional	4	11

Home and community care coordinator	5	13

Administrative staff	4	11

**CHARACTERISTICS OF PARENT CAREGIVERS AND THEIR CHILDREN WITH MEDICAL COMPLEXITY**	***(N = 10)***	**%**

***Of the Parents Gender***		

Female	9	90

Male	1	10

Home setting		

Urban	5	50

Rural	5	50

Education level		

Some post-secondary	2	20

Completed secondary/high school	1	10

Completed post-secondary	7	70

Family structure		

Never married- single parent	3	30

Married- dual parent	6	60

Divorced- single parent	1	10

***Of the Parent’s Child with Medical Complexity***		

Age in months, median (IQR)	28.5 (99.75)	

Gender		

Male	5	50

Female	5	50

Primary diagnoses		

Neurologic	4	40

Congenital/Genetic defect	3	30

Malignancy	2	20

Miscellaneous/Not elsewhere classified	1	10

# of diagnoses, mean (SD)	5.9 (2.0)	

Medications used, mean (SD)	4.7 (2.5)	

Technology devices used, mean (SD)	2.4 (1.5)	

Hospital outpatient visits, mean (SD)	14 (17.0)	


Characteristics of key informants, parent caregivers and their children with medical complexity, n (%) unless otherwise stated. * Key Informants include clinical and administrative leads of regional hub sites, and ex officio members.

The effectiveness of complex interventions, as well as their ability to reach relevant populations, is heavily influenced by the contextual elements involved in implementation [18]. The Context and Implementation of Complex Interventions (CICI) framework was utilized to demonstrate how data from our study participants corresponded to (the most relevant) four of the seven CICI framework domains: 1) Geographical; 2) Political; 3) Socio-cultural; and 4) Socio-economic. ***Table 2*** comprises the key components of the context with the domains, sub-domains and key elements identified by the key informants.

**Table 2:** Existing and required elements within each subdomain of the geographical, political, socio-cultural, and socio-economic CICI domains.


DOMAINS	SUB-DOMAINS	EXISTING ELEMENTS	REQUIRED ELEMENTS

**1.0** Geographical	**1.1** Infrastructure **1.2** Access to specialized community services	Utilizing existing physical infrastructure (i.e., children’s rehabilitation institutions)	Availability of specialized clinic space Consistent resourcing and access to specialized services between rural and urban regions Help with workforce challenges outside of major urban centres

**2.0** Political	**2.1** Project governance **2.2** Cross-regional community of practice **2.3** Resources **2.4** Teamwork	Strong provincial policy foundation and support by the Provincial Council of Maternal and Child Health (PCMCH) Established regions able to share experiences with newer regions Leadership Table facilitates cross-regional collaboration Quarterly Leadership Table meetings Complex Care Kids in Ontario (CCKO) funded as a time-limited pilot project with concurrent evaluation of its effectiveness Partnerships with home and community care services and children’s rehabilitation institutions Effective teamwork between members of the care team	Greater policy-level integration of the health and social care systems that CMC and their families frequently interface Integration between the hospital and community care sector Consistent resourcing and access to specialized services Secure and sufficient funding for clinic, home and community care Additional funding for allied health professionals and multidisciplinary team dedicated to complex care Staff recruitment and retention across all positions

**3.0** Socio-cultural	**3.1** Ideas and values shared among members **3.2** Relationships/Partnerships **3.3** Coordination/involvement with community	Relationships between care sectors to facilitate information sharing Regular steering committee meetings between sites Knowledge sharing between regions and care settings Strong family involvement during program design and dissemination	Consistent approach to integrating cross-sectoral services Role clarity between community providers and the complex care team Need to understand each team member’s role across the care continuum Greater buy-in from community providers More mental health supports for families Additional video conferencing technologies available at community clinics

**4.0** Socio-economic	**4.1** Social resources **4.2** Economic resources	Family contributions to clinic expansion and securing clinic funding through advocacy work Leveraging existing public infrastructure to run complex care clinics Disparities between urban centres and remote parts of the province for accessing resources and services Financial burdens and out-of-pocket expenses for families	Additional resources for families to receive therapies not covered through public sector (i.e., behavioural therapies)


### Mechanisms of impact

Through qualitative investigation of the experiences and perspectives of healthcare professionals involved in CCKO’s implementation, and of families who received the CCKO intervention, we explored the causal mechanisms and intermediate processes through which the delivery of CCKO was achieved and produced its effects on CMC and their families.

Key informants perceived that CCKO’s effectiveness was attributable to four main mechanisms: 1) *Complex Care Plan*: having a well-defined process for complex care plan creation assumed by a clinical key worker with specialized knowledge about the medical conditions and systems of care; 2) *Care Coordination*: establishing clear roles for care team members and opportunities for care providers to engage in ongoing learning, mutual sharing, and collaborative problem-solving; 3) *Clinical Key Worker*: having a clinical key worker who understands the patient’s medical history and upkeeps a trusting relationship with the family; and 4) *Timely Access to Care for Acute Needs*: establishing a structured mechanism for families to access timely and reliable medical assessment and guidance should urgent situations arise.

#### Complex Care Plan

The complex care plan is the executive summary of all active healthcare issues, designed to meet the child’s and their family’s goals and optimize health outcomes. As the complex care plan was the central component of the CCKO intervention for facilitating communication and exchange of information pertinent to the holistic care of a CMC amongst multiple providers, the thoughtful creation and management of this shared care planning tool was an important mechanism for improving quality of care. When asked about the development of the complex care plan, one nurse practitioner described that care plans were created by compiling both “*subjective and objective data*” from the patient’s physical assessment and their charts at various centres where care was received. The complex care plan is a “*fluid document*” that is regularly updated with every clinic visit and with family/healthcare provider input.

Since there was no systematic integration of the patient’s health records at various providers and care delivery sites, the complex care plan development called for a clinical key worker (usually a nurse practitioner) with specialized knowledge and comprehensive understanding of multiple domains of care to be able to extract relevant information from multiple sources of patient health records to compile a comprehensive shared care plan that was then reviewed with the family. Without real-time access to the complex care plan “living document” by various care providers from different settings, it became vitally important to ensure that the complex care plan was updated periodically (i.e., every 3–6 months) and that families could access and share it with all providers and services in their circle of care. A parent caregiver discussed her experience with using the complex care plan during specialist visits and in finding a private physiotherapist who could understand her child’s medical complexity. She said, “*there are so many things in [child’s] history that I can’t remember that are in the [hospital] charts that I can’t access. And I’ve done a pretty good job of keeping track of what that is, but it’s amazing what you forget… [The care plan] is a nice little format. It’s got all the people listed. If [specialists] have questions, they can go right to [the nurse practitioner]*.”

From families’ perspective, having a centralized care planning document that encompassed all the patient’s services, care needs, medical history, and key contacts gave families greater peace of mind, a sense of competence, and better care experience when interacting with new services and providers.

#### Care Coordination

In this model, an integrated service delivery team coordinates care, provides consultation and enables providers to assume collaborative care management of CMC in community and primary care settings closer to home. The CCKO intervention provides intensive care coordination for CMC that is led by a nurse practitioner. To ensure a continuous and holistic care experience for families, it was important to provide clear roles and responsibilities for each care team member, and ongoing engagement of multidisciplinary providers in learning, mutual sharing, and collaborative problem-solving in care delivery. One paediatrician described this effort as “*having all the people and programs involved in the kids’ lives, like family and the physiotherapist and the subspecialists, and the school… recognized as part of what we should be discussing in the clinic visits and focusing on all these different parts of the kid’s life and not just focusing on the medical aspect*.” As complex care patients often access multiple providers, services, and undergo transitions in care, having a team of providers each playing a distinct role in care delivery and keeping abreast of the patient’s changing conditions and needs across different systems was key for delivering care that was proactive and holistic, while minimizing errors and duplication. One parent discussed how the complex care team remained well-informed with her child’s medical situation, whereas other services sometimes had communication mix-ups. She described her complex care experience as “*you walk away feeling like they want you to succeed*.”

#### Clinical Key Worker

The CCKO intervention provides intensive care coordination for CMC that is led by a clinical key worker, usually a nurse practitioner. In addition to defining individual care team members’ roles and tasks, it was imperative to have a clinical key worker initiate and oversee the processes for mutual learning and collaborative decision-making amongst multiple providers across delivery systems, to ensure that important follow-up actions in between clinic visits are kept on track. Families’ experience of care continuity would benefit from more intentional and sustained collaboration of the complex care team with primary care physicians and emergency department providers, an aspect of care that was not consistently delivered across CCKO complex care clinics.

Having the clinical key worker role was an important mechanism through which CCKO improved patient and family outcomes. As the family’s primary point of contact, the clinical key worker built a trusting personal relationship with the family, and understood the family’s unique situation, values, preferences, and the patient’s medical history. To ensure nurse practitioners were able to satisfy their patients’ needs and meet role expectations, a caseload of 75–80 patients was recommended per 1.0 FTE. A nurse practitioner described the added value of a clinical key worker as “*helping families know that, if there is an issue, they have a go-to person that can help troubleshoot, because the journey with a child with complex medical needs can be a very scary and lonely place*.” One parent caregiver characterized their clinical key worker as a constant and reliable guide for emerging medical issues and as a knowledgeable “*higher brain*” for their family’s multisystem needs, which constituted an indispensable mechanism for making other aspects of the complex care program function as intended. Another parent caregiver said the nurse practitioner played an instrumental role in “*helping us navigate the medical system, it’s new to us and overwhelming…so just to be able to know that I don’t have to panic is phenomenal, because it just eases that anxiety*.” The relational work done by the clinical key worker was highlighted by families as an important contributor to their ability to feel supported and at ease. Therefore, some families also described how their experience of care in the CCKO program was impacted by the clinical key worker’s leave of absence and staffing turnover. Furthermore, to deliver high quality care, complex care clinics needed to have resources to support enough clinical key workers so that they each can develop trusting personal relationships with all families on their caseload.

#### Timely Access to Care for Acute Needs

Several CCKO clinics offered an on-call program in collaboration with their complex care inpatient unit to provide families with 24/7 access to an on-call paediatrician or nurse to offer timely medical evaluations to help families decide the next best step in illness management. This way, families could gain assurance and competency to effectively deal with the presenting problem, whether through going to the emergency department, visiting the clinic the next day, or self-managing at home, thereby reducing avoidable emergency visits. One paediatrician said, “*we keep all [of the patients’] information and the care plans in a binder on the ward…the patients don’t just get seen in clinic, but we also see them on the ward quite often. Some of them are ‘frequent flyers’ and being in the complex care clinic avoids them being in the emergency department*.”

## Discussion

Our study demonstrated that a strong provincial governance structure was an asset in the implementation of a new program that requires cross-regional and interorganizational partnerships, and multidisciplinary collaboration. Provincial leadership was responsible for monitoring implementation, ensuring fidelity, and recommending adjustments, whereas each of the four regional hub sites were responsible for running a tertiary complex care clinic and planning and establishing tertiary-integrated complex care clinics with community partners within their dedicated regions.

Our participants reported novel collaborations and inter-organizational partnerships between complex care teams, key community partners and regional sites, that have become stronger through CCKO’s implementation. Participants also shared how the strategy has creatively leveraged its resources and facilitated capacity building in the community to assume that care over time, by partnering with community services and giving them an equal seat at the table. Supporting collaboration between regional hub sites and children’s rehabilitation service providers to run collaborative clinics in smaller, less urbanized communities enabled CMC and their families to receive care closer to home. Consistent with other studies, our findings support the development of tertiary-based complex care programs integrated within the community as an essential way to integrate care for CMC across locations, across the care continuum and across service systems [7,20,21]. The comprehensive care that CMC and their families received includes features that appear to have contributed to improved outcomes in other studies, such as a designated key worker, 24/7 access to an on-call paediatrician or nurse, coordinated care with a multidisciplinary team-based model, timely access and reliable medical assessment and guidance when acute illness episodes arose, development of a care plan and provision of specialty services within the same clinic [22,23,24,25].

Previous literature has reported healthcare providers’ concerns regarding family capacity to navigate the system, compounded by children’s medical complexity and psychosocial complexity of the families [26]. Moreover, Cady and Belew (2017) found that parents of CMC often experience fragmented and uncoordinated communication across systems of care, where such gaps in communication were often perceived as a threat to their child’s health and wellbeing [27]. Many parents eliminate this gap by assuming responsibility for their child’s around-the-clock care and care coordination, which could lead to parental burnout [2,26,28]. The call for caregiver and parent support has been recognized by key provincial stakeholders and has remained a central tenet of the CCKO strategy, by prioritizing family involvement and engagement. Considering this, future iterations of complex care programs would find merit in attention to strengthening mental health support services for parents of CMC. Moreover, future improvements of the CCKO program would benefit from additional partnerships between health and other sectors such as education, housing, child welfare, and transportation, as these sectors have shown to provide care for children with medical complexities and their families/caregivers [29,30,31].

To enable understanding about what works in an intervention, process evaluations assess fidelity to determine whether the intervention was delivered as intended [14]. In evaluating complex integrated care interventions like the CCKO, attention to the context and circumstances surrounding the implementation was crucial for understanding how the CCKO achieved its effects, why the effects may vary, and how they can be sustained, scaled up, and adapted to other settings or populations [32]. The CCKO strategy was designed with a low fidelity implementation approach, wherein regions with notable contextual variations were encouraged to adopt various components of the CCKO model as they best saw fit and make adaptations in line with regional circumstances and capacity. The PCMCH provided support to regions in operationalizing and tailoring the CCKO model as their resources gradually expanded, provided that any adaptations remain true to the core components of CCKO (i.e., clinical key worker, complex care plan, and care coordination). However, some regions found CCKO implementation more challenging than others due to macro-level factors (e.g., resource constraints, geographical setting, personnel shortages). These challenges were more prominent in regions with newer complex care programs at the start of the CCKO roll-out. Moreover, some regions reported a lack of clear guidance for how they could adapt the CCKO care delivery model to better suit their regional and local circumstances.

In a systematic review of inter-professional collaborative interventions, Supper et al. (2015) found that a flexible model of care that was adapted for the setting and stakeholders received greater support from the care delivery team. The model of collaboration in the CCKO strategy supported this premise. Although there was a core set of common activities at each site, many of the processes and procedures conducted by healthcare professionals involved in implementing the strategy were allowed to vary depending on the context of each site and the professionals involved [33]. To achieve integrated care, an environment that fosters connectivity among healthcare providers and organizations must be created, where creative solutions will emerge through ongoing interactions over time, based on collective insights, distributed control and learning [34,35,36].

Previous research has shown that existing healthcare systems are not designed to care for patients with complex needs, especially those with chronic conditions who require care management across multiple providers and services [37]. In the context of healthcare fragmentation, there is a broad base of literature supporting the need to effectively communicate and coordinate care across sectors [38,39,40]. In our study, the fragmentation in care was translated into a disconnect in perspectives between hospital and community providers on the scope of services in each setting and provider roles. In line with process evaluations of other integrated care interventions, our findings highlight the importance of having a clear direction and communicating the intervention components to all collaborators, ensuring that all staff are trained and provided with sufficient guidance, resources and system-level support [33]. Moreover, effective interprofessional communication between all collaborators will improve the success of complex interventions involving multiple healthcare providers [33].

### Strengths, limitations, and lessons learned

Prior process evaluations of complex interventions report the MRC framework guidance as useful for better understanding the causal assumptions which underpin interventions and how interventions work in real-world contexts [33,41]. Similarly, the MRC process evaluation guidance functioned as a useful framework for reporting the process evaluation of the CCKO strategy and how it translated into practice. Using this MRC guidance, we discerned the mechanisms used to achieve the intervention outcomes, fidelity of implementation, unique contextual factors used in the rollout and tailoring of the intervention to different contexts, as well as the relationship between the components [13]. Moreover, this allowed for better understanding of how this intervention could be replicated by similar future interventions, as well as scaled up and spread in different contexts.

A strength of this study related to the inclusion of participants in diverse professions from all implementing regions who were involved in overseeing CCKO implementation and/or delivering complex care services. The study recruited participants from across the continuum of care, including participants from primary, secondary and tertiary healthcare systems, thereby ensuring the perspectives and experiences from diverse care settings, and at various program planning and implementation phases were represented. The interview guide was tailored to participants’ unique experiences with CCKO, which allowed participants some flexibility to contribute what was most important to them.

Limitations of the study include selection bias as participants were mainly, although not exclusively, from urban centres. From our findings, we can conclude that those families in remote regions experience additional barriers and likely have disproportionately lower access to resources, services, and providers, compared to those in urban centres. Moreover, the CCKO program is still in the demonstration stage, and it is still unclear if sufficient capacity exists to service all the CMC across the province with the existing resources and service delivery model. Lastly, we did not conduct a detailed economic analysis which would be helpful in better understanding of the return on investment from complex care services. Previous evaluations have reported a decrease in acute care utilization and cost from such interventions [22,42,43], but not consistently [44].

### Lessons learned

1. A strong provincial governance structure is an asset in the implementation of a new program that requires cross-regional and interorganizational partnerships, and multidisciplinary collaboration.
2. Expansion of complex care services should prioritize the building of local capacity and leveraging existing resources and infrastructure.
3. Securing sustainable funding is necessary for the longevity and expansion of the complex care intervention.
4. An adaptable and flexible approach was useful at the early implementation stage to address unique geographic, patient and family needs in various regions. However, more structure and standards are needed over time to support program stabilization.
5. Families of CMC are at a high-risk of experiencing stress and burnout. Many parent caregivers noted the significant value and utility of the CCKO intervention for reducing stress.

## Conclusion

CCKO introduced a province-wide care coordination and integration program for CMC, through institutionalizing partnerships among multiple organizations and across several care sectors. This process evaluation adds to the understanding of the processes involved in CCKO’s implementation, its mechanisms of impact and contextual factors that affected implementation success. The study highlights the work that needs to be done with families, healthcare providers, and the healthcare system to develop and sustain effective coordinated care models for CMC. Ongoing work to implement and expand models of care for CMC will benefit from enhanced access to complex care services closer to home, strong cross-regional and interorganizational partnerships, sufficient program funding, a well-trained team with expertise in complex care and family engagement throughout the program trajectory.

## Appendix: Interview guide for healthcare professionals

- What does Complex Care for Kids Ontario (CCKO) mean to you?
  – What do you think are important components of the CCKO provincial strategy?
  – What do you think are important components of the CCKO complex care delivery model?
- Can you tell me a bit about your role as it relates to the CCKO strategy to date?
- Can you describe how your organization currently delivers paediatric ambulatory complex care services?
- Have you been involved in planning and establishing the Complex Care Program at your organization *or* expanding your organization’s existing Complex Care Program? Overall, how did you find this experience?
  – What do you think may have facilitated the development of the Complex Care Program?
  – What support do you think is needed to establish a new Complex Care Program? Do you think that these supports were available during the establishment of the Complex Care Program at your organization?
  – What challenges have you encountered while establishing or expanding the Complex Care Program? Was there anything that stopped or delayed your tasks?
- The CCKO provincial strategy was launched in 2015. Since then, what changes in care delivery have there been in your organization’s Complex Care Program?
- How has your team sought feedback from families for planning or improving the Complex Care Program?
- Can you describe the process for planning and establishing new community complex care clinics integrated in your organization’s tertiary Complex Care Program?
- Can you describe your roles and responsibilities in delivering care at your organization’s paediatric Complex Care Program?
  – How does your team develop a complex care plan?
  – How do members of your team keep each other informed about complex care patients’ ongoing care and medical concerns?
  – How do you share information with other hospital departments, specialist providers, and providers from other organizations?
  – How often do you attend scheduled complex care clinic visits?
  – How often do community and specialist providers attend scheduled clinic visits?
  – What challenges have you encountered while performing your job tasks for the Complex Care Program? Was there anything that stopped or delayed your tasks?
- Overall, how did you find your experience participating in the CCKO Leadership Table?
- Which aspects of the CCKO implementation strategy do you think could have been improved?
